# Preventive effects of *Dendrobium candidum* Wall ex Lindl. on the formation of lung metastases in BALB/c mice injected with 26-M3.1 colon carcinoma cells

**DOI:** 10.3892/ol.2014.2383

**Published:** 2014-07-25

**Authors:** GUIJIE LI, PENG SUN, YALIN ZHOU, XIN ZHAO, FENG CHEN

**Affiliations:** 1 Department of Biological and Chemical Engineering, Chongqing University of Education, Chongqing 400067, P.R. China; 2 Department of Food, Nutrition and Packaging Science, Clemson University, Clemson, SC 29634, USA

**Keywords:** *Dendrobium candidum*, cytokine, anticancer, apoptosis, anti-metastasis

## Abstract

*Dendrobium candidum* Wall ex Lindl. (*D. candidum*) is a traditional Chinese medicine widely used in Asia. The present study has showed that *D. candidum* exerted an anti-metastatic effect in mice injected with 26-M3.1 colon carcinoma cells. *D. candidum* showed the most marked tumor inhibitory rate of 64.5% at a dose of 400 mg/kg body weight (b.w). The mRNA and protein expression of Bax in lung tissue of *D. candidum*-treated mice was shown to be higher as compared with control mice, whereas the mRNA and protein expression of Bcl-2 showed the opposite trend. Decreased mRNA and protein expression of MMP and increased expression of TIMPs was demonstrated in lung tissues by quantitative polymerase chain reaction and western blot assays. *D. candidum* reduced the serum cytokine levels of IL-6, IL-12, TNF-α and IFN-γ to a greater extent as compared with the control mice, and administration of 400 mg/kg b.w. resulted in a lower serum cytokine levels as compared with mice treated with 200 mg/kg b.w. Eleven compounds were in the *D. candidum* leaf, of which the functional contents may help to generate novel treatments for the prevention of lung metastases. The results of the present study have demonstrated that *D. candidum* had a potent *in vivo* antitumor and anti-metastatic effect in BALB/c mice injected with 26-M3.1 cells.

## Introduction

*Dendrobium* is a large genus of orchids, and *Dendrobium candidum* Wall. ex Lindl. (*D. candidum*) is a functional analog of *Dendrobium moniliforme* (L.) Sw. (1,2). It is a traditional Chinese medicinal herb that is used either raw or processed for healthcare products in China (3). *D. candidum* contains water-soluble polysaccharides, phenanthrenes and numerous amino acids. High contents of chrysotoxen and erianin may have inhibitory activities in liver cancer and ehrlich ascites carcinoma cells (4).

Metastasis is defined as the spread of cancer cells from one organ or area to another adjacent organ or location (5) and it is considered that malignant tumor cells have the capacity to metastasize. Cancer can occur in cells of a tissue that are genetically mutated in a progressive manner, resulting in cancer stem cells possessing a malignant phenotype (6). Metastasis is the leading cause of mortality among cancer patients, and involves the spread of cancer from a primary site and formation of new tumors in distant organs (7). Matrix metalloproteinases (MMPs) function in numerous physiological and pathological processes, including embryonic development, morphogenesis, reproduction, tissue remodeling, arthritis, cardiovascular disease and metastasis (8). MMP activity is inhibited by specific endogenous tissue inhibitors of metalloproteinases (TIMPs) (9). To prevent the majority of cancer types, improved treatments against metastasis are needed (10).

*D. candidum* has been previously shown to exhibit strong *in vitro* anticancer effects on HeLa S3 human cervical carcinoma cells and HepG2 liver cancer cells (9). In the present study, the anti-metastatic effects of *D. candidum* were investigated in mice injected with 26-M3.1 colon carcinoma cells, and the molecular mechanisms underlying the anti-metastatic effects of the *D. candidum* were studied. The *in vivo* anti-metastatic effects were determined by tumor count, cytokine levels, and mRNA and protein expression experiments. The association between the anticancer activities and functional components of *D. candidum* was additionally explored.

## Materials and methods

### Preparations of D. candidum

*D. candidum* was purchased from Shanghai Pharmacy Co., Ltd. (Shanghai, China). The *D. candidum* was stored at −80°C and freeze-dried to produce a powder. A 20-fold volume of boiling water was added to the powdered sample and extracted twice by stirring overnight. The aqueous extract was evaporated and concentrated using a rotary evaporator (N-1100; Eyela, Tokyo, Japan).

### Anti-metastatic effects of D. candidum in mice bearing 26 M3.1 cells

The following experiment was performed according to the methods of a previous study (11). 26-M3.1 colon carcinoma cells were obtained from Professor Yoon (Department of Food and Nutrition, Yuhan University, Bucheon, South Korea). The metastatic cells were cultured in Eagle’s minimum essential medium (Gibco-BRL, Carlsbad, CA, USA) supplemented with 7.5% FBS (fetal bovine serum), a vitamin solution, sodium pyruvate, non-essential amino acids and L-glutamine (Gibco-BRL) by 5% CO_2_ at 37°C. The 6-week-old female Balb/c mice (Experimental Animal Center of Chongqing Medical University, Chongqing, China) were induced lung metastasis by injecting colon 26-M3.1 cells. The experimental mice were divided three groups, there were 20 mice in each group. The control group of mice was without any treatment for 2 weeks. The *D. candidum* group mice were treated with *D. candidum* aqueous extract solutions (200 and 400 mg/kg b.w.) by gavage for 2 weeks. After 2 weeks, all the mice were intravenously inoculated with 26-M3.1 cells at the concentration of 2.5×10^4^/mouse. Two days later, the mice were sacrificed and the lungs of 10 mice in each group were fixed in Bouin’s solution (saturated picric acid: formalin: acetic acid; 15:5:1, v/v/v) (12). Then the rates of metastasis were determined by counting tumor colonies in the photos (Canon D550; Canon, Tokyo, Japan). Inhibitory rate = [(lung tumor number of control mice - lung tumor number of D. candidum treated mice)/lung tumor number of control mice] × 100%. The other lungs of 10 mice in each group were tested for reverse transcription (RT)-PCR and western blot assays. The protocol for these experiments was approved by the Animal Ethics Committee of Chongqing Medical University.

### Analysis of IL-6, IL-12, TNF-α and IFN-γ cytokines in serum by ELISA

For the serum cytokine assay, blood from the inferior vena cava was collected in a tube and centrifuged at 1100 × g and 4°C for 10 min. The serum was aspirated and assayed as described below. Concentrations of cytokines of IL-6, IL-12, TNF-α and IFN-γ in serum were measured using mouse IL-6, IL-12, TNF-α and IFN-γ ELISA kits according to the manufacturer’s instructions (Biolegend, San Diego, CA, USA). Briefly, biotinylated antibody reagent was added to 96-well plates, then supernatants of homogenized serum were added and the plates were incubated at 37°C in CO_2_ for 2 h. After washing with phosphate-buffered saline (PBS), streptavidin-horseradish peroxidase (HRP) solution was added and the plate was incubated for 30 min at room temperature. The absorbance was measured at 450 nm using a microplate reader (iMark; Bio-Rad, Hercules, CA, USA) (13).

### RT-PCR

RT-PCR was performed according to the methods descirbed in a previous study (11). The RNA of lung tissue was treated with TRIzol reagent (Invitrogen Life Technologies, Carlsbad, CA, USA) for extraction. After total RNA was digested with RNase-free DNase (Roche, Diagnostics Basel, Switzerland) for 15 min at 37°C, then the digested RNA was purified by RNeasy kit (Qiagen, Hilden, Germany). The cDNA was synthesized from 2 μg of total RNA at 37°C for l h with AMV reverse transcriptase (GE Healthcare, Little Chalfont, Buckinghamshire, UK) (2). Sequences of Bax, Bcl-2, MMPs and TIMPs were specifically amplified (Table I) by thermal cycler (Eppendorf, Hamburg, Germany). The PCR products were separated in 1.0% agarose gels and visualized with ethidium bromide staining.

### Protein extraction and western blot analysis in the gastric tissue

Total lung tissue protein was obtained with radioimmunoprecipitation assay buffer as described (12). Protein concentrations were determined with a Bio-Rad protein assay kit (Bio-Rad, Hercules, CA, USA). For the western blot analysis, aliquots of the lysate containing 30–50 μg protein were separated by SDS-PAGE and then electrotransferred onto a nitrocellulose membrane (Schleicher and Schuell, Keene, NH, USA). The membranes were subjected to immunoblot analysis and the proteins were visualized by an enhanced chemiluminescence (ECL) method (Amersham ECL GST Western Blotting Detection kit, product code: RPN1237; GE Healthcare) then transferred onto a polyvinylidene fluoride membrane (GE Healthcare), prior to blocking with 5% non-fat milk and hybridization with primary antibodies (diluted 1:1,000). The antibodies against Bax, Bcl-2, MMPs and TIMPs were obtained from Santa Cruz Biotechnology, Inc. (Santa Cruz, CA, USA). The membranes were then incubated with a horseradish peroxidase-conjugated secondary antibody (Santa Cruz Biotechnology, Inc.) for 1 h at room temperature. The blots were washed three times with PBS-Tween and then developed by ECL using GE Healthcare ECL prime western blotting detection reagent (GE Healthcare).

### Component analysis by nuclear magnetic resonance (NMR)

Dried *D. candidum* was refluxed and extracted three times with 10 times amount of ethyl acetate. The ethyl acetate extract was obtained after 1 h for every reflux extraction and decompression concentrating extraction. The total ethyl acetate extract was extracted by anhydrous ethanol three times. The ethanol extract thus produced was resuspended in water before extraction by petroleum ether, chloroform and butanol, respectively, as follows. Firstly, the ethyl acetate extract was treated by gradient elution in a silica gel column with a petroleum ether-ethyl acetate system. Secondly, the chloroform extract was treated by gradient elution in a silica gel column with a petroleum chloroform-methanol system. Finally, the butanol extract was agitated by water ultrasonic, filtered and then eluted by an HP2MGL macroporous resin column with water, 10% ethanol, 30% ethanol and 60% ethanol, respectively. Following elution, the different solvents had eluted various compounds, and their composition could be identified by NMR (Varian INOVO 400; Varian Inc., Palo Alto, CA, USA). The NMR was set at an ^1^H frequency of 300 MHz, temperature of 25°C, pulse length of 8 μsec and spin speed of 20 Hz, and scanned 64 times. The ^1^H-NMR spectra were recorded using a standard high-resolution magicangle spinning probe with magic-angle gradient.

### Statistical analysis

Data are presented as the means ± standard deviation. Differences between the mean values for individual groups were assessed by one-way analysis of variance with Duncan’s multiple range test. P<0.05 was considered to indicate a statistically significant difference. SAS, version 9.1 (SAS Institute, Inc., Cary, NC, USA) was used for statistical analyses.

## Results

### In vivo anti-metastatic effects of D. candidum

**Figure 1:**
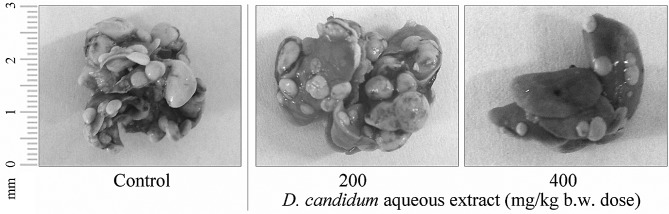
Inhibitory effects of different concentrations of *Dendrobium candidum* aqueous extract on the metastasis of tumors produced by colon 26-M3.1 cells in Balb/c mice.

26-M3.1 colon carcinoma cells were used to evaluate the anti-metastatic effects of *D. candidum in vivo*. Prophylactic inhibition of tumor metastasis by *D. candidum* was evaluated by using an experimental mouse metastasis model (Fig. 1). *D. candidum-*treated mice had significantly fewer lung metastatic colonies as compared with control mice (number of metastatic tumors, 62±6; P<0.05). A dose of 400 mg/kg b.w. *D. candidum* was the most effective at inhibiting lung metastasis. This concentration (inhibitory rate, 64.5%; number of metastatic tumors, 22±3) inhibited tumor formation and lung metastasis to a greater degree as compared with a dose of 200 mg/kg solution (inhibitory rate, 46.8%; number of metastatic tumors, 33±4).

### IL-6, IL-12, TNF-α and IFN-γ serum levels in mice

The control mice showed the highest serum levels of IL-6, IL-12, TNF-α and IFN-γ. These levels were significantly decreased in *D. candidum*-treated mice (P<0.05, Table II). A higher concentration of 400 mg/kg b.w. *D. candidum* was more effective as compared with the 200 mg/kg b.w. dose in promoting a decrease in cytokine serum levels.

### Apoptosis-related gene expression of Bax and Bcl-2 in the lung

**Figure 2:**
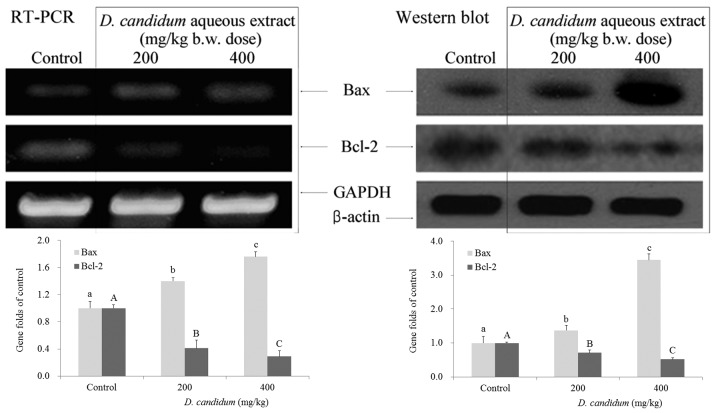
Effects of aqueous extract from *D. candidum* on the mRNA (left) and protein (right) expression of Bax and Bcl-2 in mice. Fold ratio = gene expression/GAPDH × control numerical value (control fold ratio: 1). ^a–c,A–C^Mean values with different letters over the bars are significantly different (P<0.05) according to Duncan’s multiple-range test. ^a,A^P<0.05, vs. the control; ^b,B^P<0.05, vs. the 200 mg/kg *D. candidum* dose; ^c,C^P<0.05, vs. the 400 mg/kg *D. candidum* dose. *D. candidum, Dendrobium candidum;* RT-PCR, quantitative polymerase chain reaction.

To determine which apoptotic pathways were induced by *D. candidum*, the mice were treated with 200 and 400 mg/kg b.w. dose, and the lung tissues were dissected and analyzed for apoptosis-related gene expression by RT-PCR and western blotting. As shown in Fig. 2, in the presence of *D. candidum*, there were significant differences (P<0.05) in the expression of Bax and Bcl-2, with an increase in Bax expression and a reduction in Bcl-2 expression, as determined by RT-PCR. The Bax gene expression increased with *D. candidium* treatment, in a dose dependent manner, and Bcl-2 gene expression showed a crosscurrent when mice were treated with *D. candidium* (P<0.05). The results suggested that *D. candidum* induced apoptosis in 26-M3.1 cell-injected lung metastatic mice through a Bax- and Bcl-2-dependent pathway. The increased expression of Bax and decreased expression of Bcl-2 induced by 400 mg/kg *D. candidum* was more notable at the mRNA expression level, as compared with the 200 mg/kg dose. From these results, *D. candidum* showed good anticancer effects in its ability to induce apoptosis, and these effects were observed in a dose-dependent manner.

### Metastasis-related gene expression of MMPs and TIMPs in the lung

**Figure 3:**
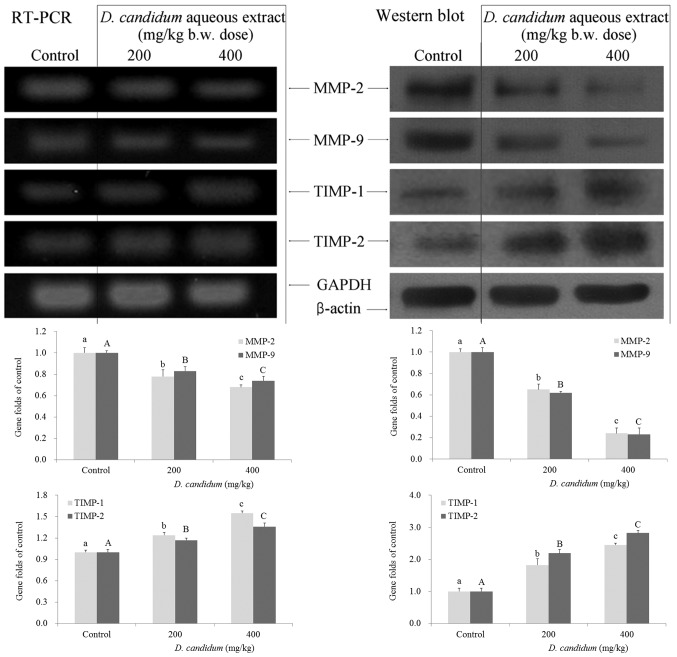
Effects of aqueous extract from *D. candidum* on the mRNA (left) and protein (right) expression of MMPs and TIMPs in mice. Fold ratio = gene expression/GAPDH × control numerical value (control fold ratio: 1). ^a–c,A–C^Mean values with different letters over the bars are significantly different (P<0.05) according to Duncan’s multiple-range test. ^a,A^P<0.05, vs. the control; ^b,B^P<0.05, vs. the 200 mg/kg *D. candidum* dose; ^c,C^P<0.05, vs. the 400 mg/kg *D. candidum* dose. *D. candidum, Dendrobium candidum;* MMP, matrix metalloproteinase; TIMP, tissue inhibitor of matrix metalloproteinases; RT-PCR, quantitative polymerase chain reaction.

RT-PCR and western blot analysis was conducted to investigate whether the inhibitory effects of *D. candidum* on metastasis were due to gene regulation of metastatic mediators, such as MMPs (MMP-2 and MMP-9) and TIMPs (TIMP-1 and TIMP-2). The expression of MMPs and TIMPs was therefore analyzed in lung tissues taken from control and *D. candidum*-treated mice. As shown in Fig. 3, *D. candidum* significantly decreased the mRNA and protein expression of MMP-2 and MMP-9 (P<0.05), and significantly increased the expression of TIMP-1 and TIMP-2 (P<0.05). The most prominent anti-metastatic effects were associated with the most marked decrease in expression of MMP-2 and MMP-9, together with the most marked increase in expression of TIMP-1 and TIMP-2. These results suggested that the higher 400 mg/kg dose of *D. candidum,* cultivated in the presence of sulfur, could elicit a stronger anti-metastatic activity as compared with the lower 200 mg/kg dose.

### Content of the D. candidum leaf

Eleven compounds were isolated and identified from the *D. candidum* leaf. Compound 1 was obtained as a clear crystal, and the ^1^H-NMR spectrum of this compound was as follows: δ 6.92 (2H, d), 6.62 (2H, d), 6.06 (2H, s), 6.03 (1H, s), 2.65 (4H, m). This compound was confirmed as dihydrogen resveratrol. Compound 2 was obtained as a white powder, and the 1H-NMR spectrum of this compound was as follows: δ 6.98 (2H, d), 6.74 (2H, d), 6.62 (1H, s), 6.47 (1H, d), 4.83 (1H,d), 4.63 (1H, d), 3.1–3.8 (12H), 3.73 (3H, s), 3.69 (3H, s), 2.74 (4H, m). This compound was confirmed as dendromoniliside E. Compound 3 was obtained as a black red needle, and the ^1^H-NMR spectrum of this compound was as follows: δ 11.00 (1H, s), 8.15 (1H, d), 6.06 (2H, s), 8.07 (1H, d), 6.95 (1H, s), 6.83 (1H, s), 6.15 (1H, s), 3.96 (3H, s), 3.93 (3H, s). This compound was confirmed as denbinobin. Compound 4 was obtained as a colorless needle, and the ^1^H-NMR spectrum of this compound was as follows: δ 4.72 (2H, m), 3.85 (1H, d), 6.06 (2H, s), 2.53 (1H, d), 2.49 (1H, t), 2.39 (1H, dd), 2.21 (1H, dd), 1.64 (1H, m), 1.35 (3H, s), 1.03 (3H, d), 0.95 (3H, d). This compound was confirmed as aduncin. Compound 5 was obtained as a white needle, and the ^1^H-NMR spectrum of this compound was as follows: δ 8.25 (1H, s), 8.10 (1H, s), 5.90 (1H, d), 4.66 (1H, dd), 3.5–4.2 (4H, m). This compound was confirmed as adenosine. Compound 6 was obtained as a white powder, and the ^1^H-NMR spectrum of this compound was as follows: δ 7.95 (1H, d), 5.85 (1H, d), 5.66 (1H, d), 3.2–4.3 (5H, m). This compound was confirmed as uridine. Compound 7 was obtained as a clear crystal, and the ^1^H-NMR spectrum of this compound was as follows: δ 10.60 (1H, s), 7.92 (1H, s), 6.45 (2H, s), 5.66 (1H, d), 3.4–4.4 (5H, m). This compound was confirmed as guanosine. Compound 8 was obtained as a white powder, and the ^1^H-NMR spectrum of this compound was as follows: δ 7.65 (1H, d), 7.41 (2H, d), 6.85 (2H, d), 6.33 (1H, d), 4.17 (2H, t), 1.69 (2H,m), 1.25 (54H, m), 0.85 (3H, t). This compound was confirmed as defuscin. Compound 9 was obtained as a white powder, and the ^1^H-NMR spectrum of this compound was as follows: δ 7.45 (2H, d), 6.82 (2H, d), 6.81 (1H, d), 5.83 (1H, d), 4.16 (2H, t), 1.67 (2H, m), 1.23 (54H, m), 0.88 (3H, t). This compound was confirmed as *n*-triacontyl *cis*-*p*-coumarate. Compound 10 was obtained as a white powder, and the ^1^H-NMR spectrum of this compound was as follows: δ 2.35 (2H, t), 1.62 (2H, m), 1.25 (24H, m), 0.88 (3H, t). This compound was confirmed as hexadecanoic acid. Compound 11 was obtained as a white powder, and the ^1^H-NMR spectrum of this compound was as follows: δ 3.85 (2H, t), 1.75 (2H, m), 1.45 (2H, m), 1.22 (54H, m), 0.85 (3H, t). This compound was confirmed as hentriacontane.

## Discussion

Although *D. candidum* has been used as a traditional Chinese medicine, there has been little scientific research regarding its mechanism of action. *D. candidum* contains high concentrations of benzenes and their derivatives, phenolic, lignans, lactone, flavonoids and 18 novel *D. candidum* pigments (14). *D. candidum* has been previously reported to have various therapeutic effects on numerous pathological conditions, including inflammation, immunity, hyperglycemia and cancer (15).

Lower levels of IL-6, IL-12, IFN-γ and TNF-α cytokines are indicative of improved anticancer effects (16,17). IL-6 is regarded as an important tumor-promoting factor in various types of human cancer. An increased expression of IL-6 has been found in patients with cancer, in serum and tumor tissue (18). IL-12 has been shown to contribute to tumor eradication, through IFN-γ-dependent induction of the anti-angiogenic factors interferon-inducible protein 10 and monokine induced by gamma interferon (19). In addition, a previous study has shown that drugs targeting TNF-α may be useful for the treatment of cancers (13). In the present study, it was observed that the levels of IL-6, IL-12, TNF-α and IFN-γ in mice injected with 26-M3.1 cells were markedly decreased following *D. candidum* treatment. Based on this study, *D. candidum* showed a strong preventive effect on the development of lung metastases.

Tumor cells are able to migrate to another site, penetrate the vessel walls, continue to multiply and eventually form another tumor. Colon 26-M3.1 carcinoma cells have been previously used to evaluate anti-metastatic effects *in vivo* (20). Based on *in vivo* data from previous studies, 26-M3.1 colon carcinoma cells were used to examine the effects of *D. candidum* on metastasis in mice. The results further proved the activity of *D. candidum*, and the observed anticancer effects occurred in a dose-dependent manner.

Apoptosis is a fundamental cell event, and understanding its mechanisms of action will have a significant effect on antitumor therapy. The Bcl-2 family, which includes promoters (Bax and Bid) and inhibitors (Bcl-2 and Bcl-xL), is a key regulator in mitochondria-mediated apoptosis (21). In the present study, the gene and protein expression of Bax was increased, whereas the protein expression of Bcl-2 was decreased following treatment with *D. candidum*. Based on the gene expression results, *D. candidum* showed strong activity in promoting apoptosis in cancer.

MMPs comprise a family of zinc-dependent endopeptidases that function in tumorigenesis and metastasis (22). MMPs can cleave the majority of all extracellular matrix (ECM) substrates. Degradation of the ECM is a key event in tumor progression, invasion and metastasis (23). Among the MMP family members, MMP-2 and MMP-9 are molecules important for cancer invasion, and have been shown to be highly expressed in cancer cells (24). Inhibition of MMP activity is useful for controlling tumorigenesis and metastasis (25). TIMPs are naturally occurring inhibitors of MMPs that prevent catalytic activity by binding to activated MMPs, thereby blocking the degradation of the ECM. Disturbances in the ratio between MMPs and TIMPs have been observed during tumorigenesis (26). Maintaining the balance between MMPs and TIMPs, or increasing TIMP activity, are useful methods by which to control tumor metastasis (27). In the present study, strong anti-metastatic effects were correlated with a reduction of MMPs and an increase of TIMPs following administration of *D. candidum* in mice. From the results, *D. candidum* showed a strong anti-metastatic effect and, therefore, may be a functional drug for cancer prevention.

Numerous compounds were isolated and identified by NMR of the *D. candidum* leaf in the present study. Resveratrol is an antioxidant that has been often recommended for use as treatment in patients with colon cancer (28). Denbinobin is a biologically active chemical that has been demonstrated to inhibit colon cancer growth both *in vitro* and *in vivo* (29). Aduncin is a unique component that has only been found in Dendrobium. Aduncin may have anticancer effects, but its function requires further research (30). Adenosine serves as a physiological regulator and acts as a cardio-, neuro- and chemo-protector, and as an immunomodulator. Adenosine has been shown to exert anticancer effects at certain concentration. When administered in combination with chemotherapy, adenosine can enhance the chemotherapeutic index and acts as a chemoprotective agent (31). The availability of uridine can alter the sensitivity of tumor cells to antimetabolites. Adenine is incorporated into polynucleotides and the two compounds have been identified as important cancer-associated substances (32). Defuscin, *n*-triacontyl *cis*-*p*-coumarate, hexadecanoic acid and hentriacontane have also shown functional activities in human health (33,34). Taken together, these compounds all exhibit anticancer activities, and with the high content of functional compounds, this may explain why *D. candidum* showed a functional effect in cancer prevention. The synergy of these bioactive components may increase the anticancer effects of *D. candidum*.

In summary, the present study found that *D. candidum* has potent *in vivo* anti-metastatic activity, particularly in the inhibition of *in vivo* tumor metastasis. The analysis of cytokine levels, as well as mRNA and protein expression, has provided a mechanistic basis for these functional effects and a scientific basis for the development of *D. candidum* in cancer therapy.

## Figures and Tables

**Table I:** Primer sequences used for quantitative polymerase chain reaction.

Gene name	Sequence
Bax	
Forward	5′-AAGCTGAGCGAGTGTCTCCGGCG-3′
Reverse	5′-CAGATGCCGGTTCAGGTACTCAGTC-3′
Bcl-2	
Forward	5′-CTCGTCGCTACCGTCGTGACTTGG-3′
Reverse	5′-CAGATGCCGGTTCAGGTACTCAGTC-3′
MMP-2	
Forward	5′-CTTCTTCAAGGACCGGTTCA-3′
Reverse	5′-GCTGGCTGAGTACCAGTA-3′
MMP-9	
Forward	5′-TGGGCTACGTGACCTATGAC-3′
Reverse	5′-GCCCAGCCCACCTCCACTCC-3′
TIMP-1	
Forward	5′-GTCAGTGAGAAGCAAGTCGA-3′
Reverse	5′-ATGTTCTTCTCTGTGACCCA-3′
TIMP-2	
Forward	5′-TGGGGACACCAGAAGTCAAC-3′
Reverse	5′-TTTTCAGAGCCTTGGAGGAG-3′
GAPDH	
Forward	5′-CGGAGTCAACGGATTTGGTC-3′
Reverse	5′-AGCCTTCTCCATGGTCGTGA-3′

**Table II:** Serum IL-6, IL-12, TNF-α, and IFN-γ levels of mice bearing 26-M3.1 cells treated with *D. candidum.*

Treatment	IL-6	IL-12	TNF-α	IFN-γ
Control (untreated)	235.7±33.8^a^	712.1±55.8^a^	84.1±12.0^a^	80.5±7.7^a^
*D. candidum (*mg/kg)				
200	172.7±21.6^b^	571.7±42.9^b^	61.2±4.9^b^	63.8±5.9^b^
400	113.0±26.1^c^	338.6±39.7^c^	50.6±6.6^c^	44.8±6.2^c^

a–c Mean values with different letters in the same column are significantly different (P<0.05) according to Duncan’s multiple range test.

D. candidum, Dendrobium candidum.
